# Efficacy and safety of immune checkpoint inhibitors with or without radiotherapy in metastatic non-small cell lung cancer: A systematic review and meta-analysis

**DOI:** 10.3389/fphar.2023.1064227

**Published:** 2023-01-24

**Authors:** Zijing Liu, Tiankai Xu, Pengyu Chang, Weijia Fu, Jiaying Wei, Chengcheng Xia, Qiang Wang, Man Li, Xiaoyu Pu, Fuxue Huang, Chao Ge, Yan Gao, Shouliang Gong, Chengjiang Liu, Lihua Dong

**Affiliations:** ^1^ Jilin Provincial Key Laboratory of Radiation Oncology and Therapy, The First Hospital of Jilin University, Changchun, China; ^2^ Department of Radiation Oncology, The First Hospital of Jilin University, Changchun, China; ^3^ NHC Key Laboratory of Radiobiology, School of Public Health of Jilin University, Changchun, China; ^4^ Department of Gastroenterology/General Practice, Anhui Medical University, He Fei, China

**Keywords:** radiotherapy, immune checkpoint inhibitors, non-small cell lung cancer, safety, efficacy

## Abstract

**Background and purpose:** Although immune checkpoint inhibitors (ICIs) have become the first-line treatment for metastatic non-small cell lung cancer (mNSCLC), their efficacy is limited. Meanwhile, recent reports suggest that radiotherapy (RT) can activate the systemic antitumor immune response by increasing the release of antigens from tumor tissues. Therefore, in patients with mNSCLC treated with ICIs, investigations were performed to determine whether the addition of RT improved the outcomes. Furthermore, the adverse events rate was evaluated.

**Methods and materials:** Pubmed, Embase, and Cochrane Library were searched using the keywords “radiotherapy,” “immune checkpoint inhibitors,” and “non-small cell lung cancer” from the date of inception to 2 May 2022. Randomized controlled trials (RCTs) and nonRCTs (NRCTs) comparing the efficacy and safety of RT combined with ICIs *versus* ICIs alone in metastatic NSCLC were assessed. The primary outcomes were progression-free survival (PFS) and overall survival (OS), and the secondary outcomes were abscopal response rate (ARR), abscopal control rate (ACR), adverse events rate, and pneumonia rate. The analyses were conducted using the Mantel–Haenszel fixed-effects or random-effects model. The I^2^ statistic was used to determine heterogeneity, whereas funnel plots and Egger’s test were used to assess publication bias.

**Results:** In 15 clinical studies, 713 patients received RT combined with ICIs and 1,275 patients received only ICIs. With regard to PFS and OS, the hazard ratios of RT combined with ICIs were 0.79 (0.70, 0.89) and 0.72 (0.63, 0.82), respectively. In terms of ARR and ACR, the odds ratios (ORs) of RT combined with ICIs were 1.94 (1.19, 3.17) and 1.79 (1.08, 2.97), respectively. Subgroup analyses based on study type (RCT/NRCT), RT target (intracranial/extracranial), number of RT sites (single site), previous ICI resistance (yes/no), and sequencing of RT and ICIs (concurrent/post-RT ICIs) revealed that the addition of RT significantly prolonged PFS and OS. However, subgroup analyses based on radiation dose/fractionation indicated that the addition of hypofractionated RT significantly prolonged OS but not PFS. When grouped according to the level of PD-L1 expression, the addition of RT prolonged PFS only in patients who were PD-L1-negative. Furthermore, subgroup analyses of ARR and ACR signified that the combination therapy resulted in better local control of lesions outside the irradiation field in the hypofractionated RT, extracranial RT, and ICI-naïve subgroups. In terms of adverse events, the addition of RT did not significantly increase the adverse events rate but was associated with a higher pneumonia rate [OR values were 1.24 (0.92, 1.67) and 1.76 (1.12, 2.77), respectively].

**Conclusion:** Meta-analysis of existing data suggests that the addition of RT can significantly prolong PFS and OS in patients with metastatic NSCLC receiving ICIs. In addition to lesions in the irradiation field, RT can improve the local control rate of lesions outside the irradiation field *via* immune activation. Combination therapy does not increase the overall risk of adverse reactions, except for pneumonia.

## 1 Introduction

According to the global epidemiological survey of malignant tumors, lung cancer has the highest mortality rate and the second-highest incidence rate among 36 malignant tumors (Bray et al., 2018; Sung et al., 2021). Of these, as many as 65.33% of men diagnosed with lung cancer were locally advanced (stage III) or had metastases (stage IV) (Chen et al., 2014; Meza et al., 2015). The two main histological subtypes are non-small cell lung cancer (NSCLC) and small cell lung cancer (SCLC), accounting for 76% and 13% of all lung cancer cases in the United States, respectively (Howlader et al., 2020).

Immune checkpoint inhibitors, including anti-PD-1/anti-PD-L1, significantly improve the rate of survival in cases with advanced NSCLC (Mok et al., 2019; Reck et al., 2019; Nishio et al., 2021). The Keynote-001 study showed that immunotherapy was well tolerated (Garon et al., 2015). However, at the same time, ICIs induce primary/secondary resistance owing to their internal and external factors, and only 17%–48% of the patients respond to immunotherapy-based approaches (Morad et al., 2021). The control of local lesions was also not objectively adequate, and the control of distant lesions was limited (Weiner, 2008; Shankar et al., 2020).

As an important treatment modality for mNSCLC, radiotherapy (RT) can enhance the systemic release of antigens from the tumor tissues. These antigens are recognized by antigen-presenting cells and are subsequently presented to T lymphocytes, especially CD8 cytotoxic T cells. The priming and activation of these cells trigger a systemic immune response against the tumor tissue, both in irradiated lesions within the irradiated field and in unirradiated lesions outside the irradiation field. In multiple murine solid tumor models, including glioma, NSCLC, and melanoma, RT combined with ICIs has demonstrated stronger antitumor effects than monotherapy (Deng et al., 2014; Twyman-Saint Victor et al., 2015; Gong et al., 2017). On the basis of ICIs, studies on various advanced solid tumors have reported that the immune activation effect of RT can improve the abscopal local control rate and survival time. The addition of RT to patients with melanoma treated using ICIs has been reported to increase abscopal local control by up to 6.5% and overall survival (OS) by 8 months. However, no significant benefits were seen in recurrent/metastatic head and neck tumors, adenoid cystic carcinoma, and small cell lung cancer (Pakkala et al., 2020; Mahmood et al., 2021; McBride et al., 2021). For mNSCLC, whether the addition of RT to ICIs can offer long-term efficacy benefits and whether it can further improve the control of abscopal local lesions is yet to be determined. Therefore, a systematic review and meta-analysis were conducted to compare the efficacy and safety of RT combined with ICIs and ICIs alone in patients with mNSCLC.

## 2 Materials and methods

We conducted a systematic review and meta-analysis following the preferred reporting items of the guidelines for systematic reviews and meta-analyses (Moher et al., 2009). The protocol for this meta-analysis is available in prospero (CRD42022327432).

### 2.1 Search trials

We searched PubMed, the Cochrane Library, and Embase databases using the keywords “radiotherapy”, “immune checkpoint inhibitors”, and “non-small cell lung cancer” according to the PICOS principles from the date of inception to 2 May 2022 in order to identify the published randomized controlled trials (RCTs) and non-RCTs (NRCTs). P: mNSCLC; I: RT combined with ICIs; C: ICIs alone; O: progression-free survival (PFS), OS, abscopal response rate (ARR), abscopal control rate (ACR), adverse events rate, and pneumonia rate; S: Studies directly comparing RT plus ICIs with ICIs alone. No language restriction was placed. We also searched the references of articles that met our inclusion criteria. Detailed search strategies are reported in Supplementary Table S1.

### 2.2 Inclusion and exclusion criteria

We included published studies that met the following criteria:

• NSCLC confirmed by histopathology.
• Disease being metastatic or stage IV disease.
• Studies directly comparing RT combined with ICIs and ICIs alone, either RCTs or NRCTs. In addition, we confirmed that RT received by the mNSCLC patients included in this study was the first RT after the diagnosis of mNSCLC.
• Quantitative meta-analysis data reported for at least one outcome measure [hazard ratio (HR) for PFS/OS or odds ratio (OR) for ARR, ACR, adverse events rate, or pneumonia rate].

Studies that did not meet these criteria were excluded, as were the studies that met the following criteria:

• Studies on malignancies other than NSCLC.
• Studies including Abstracts only (full version not available), which we believe may lead to bias.
• Ongoing research.

After searching for studies, two researchers discarded all duplicate studies obtained from various databases and independently screened the titles and abstracts of all remaining articles to exclude the manuscripts that did not meet the inclusion criteria. In addition, the researchers carefully reviewed the full text of all shortlisted articles for inclusion in this study. Finally, the third investigator discussed the controversial study with the two investigators who conducted the screening. Any disagreements regarding study eligibility were resolved through consulting with the fourth investigator.

### 2.3 Risk-of-bias assessments

The methodological quality of the included RCTs was independently assessed by two researchers (Liu Zijing and Xu Tiankai) according to the Cochrane risk of bias criteria. The 7 items used to assess bias in each trial included random sequence generation, allocation concealment, participants, and personnel, blinding of outcome assessments, complete outcome data, selective reporting, and other biases. We defined other bias as the difference in the baseline characteristics between the intervention groups. The Newcastle–Ottawa scale was used in cohort or case-control studies. The included trials were classified as either low-quality, high-quality, or moderate-quality based on the following criteria: 1) the trials were considered low-quality if randomization or allocation concealment was assessed as a high risk of bias, irrespective of the risk of other items; 2) trials were considered to be of high quality when randomization and allocation concealment were assessed as a low risk of bias and all other items were assessed as low risk of bias or unclear risk of bias in trials; 3) trials were considered to be of moderate quality if they did not meet the criteria for high risk of bias or low risk.

### 2.4 Data extraction

Two researchers independently extracted the following information from each study: major author; publication year; publication country; type of studies; the number of patients; RT target; RT technique; RT dose (Gy)/fraction; timing of RT and ICIs intervention; type of ICIs; ICIs dose. The differences were resolved by consensus. Primary outcomes were PFS and OS, while the secondary outcomes were ARR, ACR, adverse events rate, and pneumonia rate. Furthermore, as some of the data were not explicitly reported, we contacted experimental institutions to obtain as much comprehensive information as possible. If the HR values for OS and PFS were not provided in the original text, we extracted data from the survival curves using digitizing software.

### 2.5 Data analysis

We used STATA 15 (version 14.3; College Station, TX, United States) for meta-analysis and the assessment of publication bias, generation of funnel plots, and assessment of heterogeneity. We also judged the publication bias of the included studies by using a funnel plot, followed by a quantitative assessment by Egger’s test. We used the I^2^ statistic to assess heterogeneity between trials. If there was no significant heterogeneity, we pooled the data using a Mantel–Haenszel fixed-effects model (I^2^ < 50%); however, if there was significant heterogeneity (I^2^ ≥ 50%), a random-effects model was employed. Dichotomous outcomes (ARR, ACR, adverse reaction rate, and pneumonia rate) were expressed as ORs with 95% confidence intervals (CIs). Survival data (PFS, OS) were expressed as HR with 95% CI. According to the Cochrane Handbook’s recommendations for practice, trials with zero events in both the intervention and control groups were not included in the meta-analyses when calculating HR and OR. We performed sensitivity analyses as per the “leave-one-out” method, which was used to determine the effect of each study on the overall outcome by deleting each study. Each estimate and its upper and lower CIs represent the HR or OR after removing a single study. The critical value for both OR and HR was 1. The results were considered robust if the new HR or OR value was consistent with the original HR or OR value on the same side of the cut-off after excluding any single study. All tests were 2-tailed, and *p* < 0.05 was considered to indicate statistical significance.

## 3 Results

### 3.1 Retrieved studies and their characteristics

**FIGURE 1:**
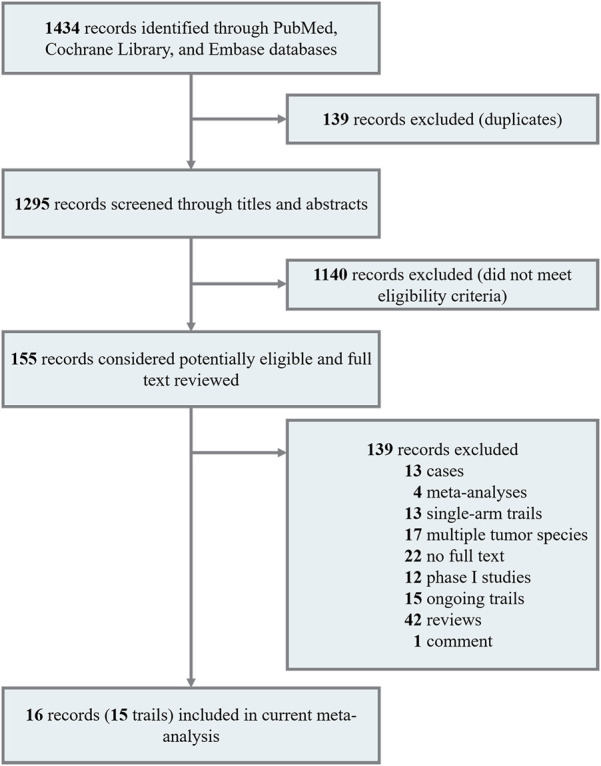
Literature search and screening process.

Our search strategy identified 1,434 records. After eliminating the duplicates, 1,295 unique records remained. Of these, 1,140 records were ineligible for inclusion, and 155 full texts were assessed for eligibility. After excluding 139 studies because the article type was not suitable for inclusion (including 13 case studies, 4 meta-analyses, 13 single-arm studies, 17 studies on other tumor types, 22 conference papers with abstracts only, 12 phase I studies, 15 ongoing studies, 42 reviews, and 1 comment), 3 RCTs and 12 NRCTs remained. A total of 15 clinical studies were reported in 16 publications (Figure 1) (Kataoka et al., 2017; Shaverdian et al., 2017; Tamiya et al., 2017; Fiorica et al., 2018; Theelen et al., 2019; Welsh et al., 2020; Hosokawa et al., 2021; Metro et al., 2021; Samuel et al., 2021; Sheng et al., 2021; Theelen et al., 2021; Öjlert Å et al., 2021; Guo et al., 2022; Qiang et al., 2022; Schoenfeld et al., 2022; Wang et al., 2022).

The RT targets for three RCTs and five NRCTs were extracranial nonbrain metastases (Tamiya et al., 2017; Fiorica et al., 2018; Theelen et al., 2019; Welsh et al., 2020; Hosokawa et al., 2021; Öjlert Å et al., 2021; Qiang et al., 2022; Schoenfeld et al., 2022). Three nonrandomized controlled studies included extracranial nonbrain metastases and intracranial brain metastases (Shaverdian et al., 2017; Samuel et al., 2021; Wang et al., 2022). In three nonrandomized controlled studies, the RT targets included only brain metastases (Metro et al., 2021; Sheng et al., 2021; Guo et al., 2022). One NRCT did not specify the radiotherapy target (Kataoka et al., 2017). Two studies by Schoenfeld and Hosokawa were on immune rechallenge after resistance to ICIs (Hosokawa et al., 2021; Schoenfeld et al., 2022). Table 1 displays the characteristics of the included studies (number of patients included, RT targets, number of RT sites, RT technique, RT dose/fraction, ICI resistance, the timing of RT and ICI intervention, ICI type, and ICI dose). Supplementary Tables S2, S3 present the quality assessment results of the included studies.

**TABLE 1:** Characteristics of the included studies.

Author	Year	Country	Study type	Patients	RT target	RT site	RT technique	RT dose/fraction	ICIs resistance	ICIs sequencing	ICIs type	ICIs dose	References
Schoenfeld	2022	America	RCT	78	MRT	Single	Low-dose RT	8Gy (0.5Gy/f, 2f/d, repeated for each of the first four 28 days cycles of therapy)	Yes	Concurrent	Durvalumab and Tremelimumab	Durvalumab (1,500 mg intravenously every 4 weeks)	Schoenfeld et al. (2022)
					TRT		Hypo-fractionated RT	24 Gy/3f				Tremelimumab (75 mg intravenously every 4 weeks)	
Welsh	2020	America	RCT	72	MRT	Single	SBRT	50 Gy/4f	No	Concurrent	Pembrolizumab	200 mg every 3 weeks	Welsh et al. (2020)
					TRT		Traditionally fractionated RT	45 Gy/15f		Sequential (salvage RT)			
Theelen	2019	Netherlands	RCT	76	MRT	Single	SBRT	24 Gy/3f	No	Concurrent	Pembrolizumab	200 mg/kg every 3 weeks	Theelen et al. (2019)
					TRT								
Shaverdian	2017	America	NRCT	97	BMRT	Not clear	Con-RT	Not clear	No	Sequential (After RT)	Pembrolizumab	2 mg/kg of bodyweight or 10 mg/kg every 3 weeks, or 10 mg/kg every 2 weeks	Shaverdian et al. (2017)
					MRT		SRS						
					TRT		SBRT						
Qiang	2022	China	NRCT	110	MRT	Multiple	Not clear	Not clear	No	Concurrent	Pembrolizumab	200 mg per intravenous infusion every 3 weeks	Qiang et al. (2022)
Wang	2021	China	NRCT	152	BMRT	Single	Not clear	Not clear	No	Concurrent	Sintilimab	Not clear	Wang et al. (2022)
					MRT						Toripalimab		
					TRT						Camrelizumab		
											Nivolumab		
											Pembrolizumab		
Samuel	2020	Australia	NRCT	269	BMRT	Single	Hypo-fractionated RT	Not clear	No	Concurrent	Pembrolizumab	Nivolumab (3 mg/kg)	Samuel et al. (2021)
					MRT		SRS				Nivolumab	Pembrolizumab	
					TRT		WBRT					200 mg intravenously	
Kataoka	2017	Japan	NRCT	146	Not clear	Not clear	Not clear	Not clear	No	Concurrent	Nivolumab	Not clear	Kataoka et al. (2017)
Hosokawa	2020	Japan	NRCT	531	TRT	Single	Not clear	Not clear	Yes	Concurrent	Not clear	Not clear	Hosokawa et al. (2021)
Fiorica	2018	Italy	NRCT	35	MRT	Single	Hypo-fractio nated RT	8–16 Gy/1-2f	No	Sequential (At least 1 week from the end of RT)	Nivolumab	3 mg/kg dose every 2 weeks	Fiorica et al. (2018)
					TRT			36 Gy/12f					
Öjlert	2021	Norway	NRCT	78	MRT	Not clear	Not clear	Not clear	No	Sequential (After RT)	Not clear	Not clear	Öjlert Å et al. (2021)
					TRT								
Tamiya	2017	Japan	NRCT	201	TRT	Single	Not clear	Not clear	No	Sequential (After RT)	Nivolumab	3 mg/kg intravenously every 2 weeks	Tamiya et al. (2017)
Sheng	2021	China	NRCT	41	BMRT	Not clear	WBRT	Not clear	No	Concurrent	Pembrolizumab	Not clear	Sheng et al. (2021)
							SRS				Nivolumab		
											Sintilimab		
											Atezolizumab		
Metro	2021	Italy	NRCT	30	BMRT	Not clear	WBRT	Not clear	No	Sequential (After RT)	Pembrolizumab	Dose of 200 mg every 3 weeks	Metro et al. (2021)
							SRS						
Guo	2022	China	NRCT	461	BMRT	Not clear	WBRT	WBRT (30–40 Gy/10–20f) SRS (15–24 Gy/1f, 20–30 Gy/3f, 24–32 Gy/4f, and 25–35 Gy/5f)	No	Concurrent	Pembrolizumab	Not clear	Guo et al. (2022)
							SRS				Nivolumab		
											Camrelizumab		
											Atezolizumab		

Abbreviations: RT, radiotherapy; ICIs, Immune checkpoint inhibitors; RCT, randomized controlled trial; NRCT, Non-randomized controlled trial; TRT, thoracic radiotherapy; MRT, radiotherapy of metastases; BMRT, brain metastases radiotherapy; SBRT, stereotactic body radiation therapy; SRS, stereotactic radiosurgery; WBRT, whole brain radiotherapy.

### 3.2 RT plus ICIs *versus* ICIs alone: Long-term efficacy and abscopal local control of lesions

**FIGURE 2:**
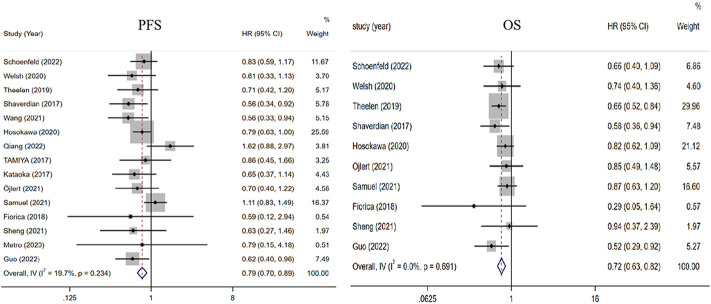
Forest plot of the survival benefits associated with RT combined with ICIs and ICIs alone. In terms of PFS and OS, the HRs of RT combined with ICIs were 0.79 (0.70, 0.89) and 0.72 (0.63, 0.82), respectively. Abbreviations: RT, radiotherapy; ICIs, immune checkpoint inhibitors; PFS, progression-free survival; OS, overall survival; HR, Hazard ratio.

All 15 studies comparing the benefit of RT plus ICIs with ICI alone in PFS were included (Kataoka et al., 2017; Shaverdian et al., 2017; Tamiya et al., 2017; Fiorica et al., 2018; Theelen et al., 2019; Welsh et al., 2020; Hosokawa et al., 2021; Metro et al., 2021; Samuel et al., 2021; Sheng et al., 2021; Öjlert Å et al., 2021; Guo et al., 2022; Qiang et al., 2022; Schoenfeld et al., 2022; Wang et al., 2022). Compared with ICIs alone, the HR of RT plus ICIs was 0.79 (0.70, 0.89, *p* < 0.001). Ten studies that compared the OS of RT plus ICIs with ICIs alone were included (Shaverdian et al., 2017; Fiorica et al., 2018; Theelen et al., 2019; Welsh et al., 2020; Hosokawa et al., 2021; Samuel et al., 2021; Sheng et al., 2021; Öjlert Å et al., 2021; Guo et al., 2022; Schoenfeld et al., 2022). Compared with ICIs alone, the HR of RT plus ICIs was 0.72 (0.63, 0.82, *p* < 0.001). The heterogeneity trend was low in PFS (I^2^ was 19.7%) and absent in OS (I^2^ was 0.0%) (Figure 2).

**FIGURE 3:**
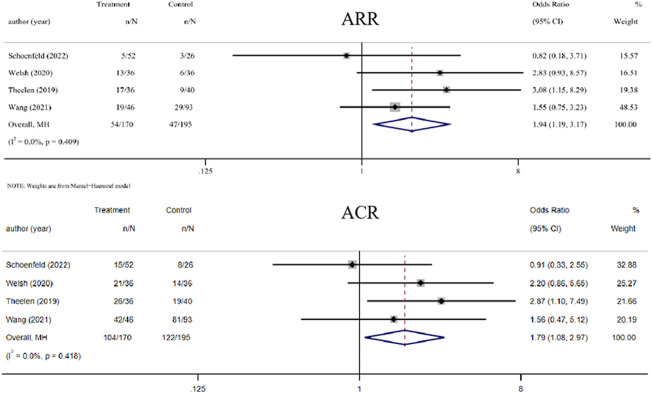
Forest plot of abscopal local control beyond the irradiation field associated with radiotherapy combined with ICIs *versus* ICIs alone. In terms of ARR and ACR, the ORs of RT combined with ICIs treatment were 1.94 (1.19, 3.17) and 1.79 (1.08, 2.97), respectively. Abbreviations: RT, radiotherapy; ICIs, immune checkpoint inhibitors; OR, odds ratio, ARR, abscopal response rate; ACR, abscopal control rate.

Four trials compared the ability of RT plus ICIs to control abscopal lesion outside the irradiation field with that of ICIs alone (Theelen et al., 2019; Welsh et al., 2020; Schoenfeld et al., 2022; Wang et al., 2022). Compared with ICIs alone, the ORs of ARR and ACR of RT plus ICIs were 1.94 (1.19, 3.17, *p* = 0.008) and 1.79 (1.08, 2.97, *p* = 0.024), respectively. Furthermore, there was no heterogeneity in the results (I^2^ was 0.0% for both) (Figure 3).

Subgroup analyses were performed according to the study type. The HRs of PFS for RCT and NRCT were 0.75 (0.58, 0.98, *p* = 0.035) and 0.80 (0.70, 0.91, *p* = 0.001), respectively. The HRs of OS for RCT and NRCT were 0.67 (0.55, 0.82, *p* < 0.001) and 0.76 (0.64, 0.91, *p* = 0.002), respectively. Heterogeneity was lacking in PFS and OS in RCTs and in OS in non-RCTs (I^2^ was 0.0% for both), unless RCTs had moderate heterogeneity in PFS (I^2^ = 33.3%) (Tables 2, 3).

**TABLE 2:** Subgroup analysis of PFS and OS of RT combined with ICIs *versus* ICIs alone in mNSCLC.

Variable	No. of trials	PFS			No. of trials	OS		
		HR (95% CI)	I^2^ (%)	*p*-value		HR (95% CI)	I^2^ (%)	*p*-value
Type								
RCT	3	0.75 (0.58, 0.98)	0.0	0.035	3	0.67 (0.55, 0.82)	0.0	<0.001
NRCT	12	0.80 (0.70, 0.91)	33.3	0.001	7	0.76 (0.64, 0.91)	0.0	0.002
RT technique								
Hypofractionated RT	6	0.95 (0.77, 1.18)	0.0	0.657	3	0.66 (0.52, 0.82)	0.0	<0.001
Hyperfractionated RT	1	0.83 (0.50, 1.38)	0.0	0.472	1	0.61 (0.30, 1.23)	0.0	0.167
Traditionally fractionated RT	1	0.91 (0.45, 1.85)	0.0	0.794	0	Not estimable	NA	NA
RT target								
Incranial RT	4	0.55 (0.39, 0.79)	18.3	0.001	2	0.61 (0.38, 1.00)	10.8	0.048
Excranial RT	10	0.76 (0.66, 0.88)	20.3	<0.001	6	0.72 (0.62, 0.84)	0.0	<0.001
RT site								
Single	8	0.82 (0.72, 0.95)	9.2	0.006	6	0.74 (0.64, 0.86)	0.0	<0.001
Multiple	1	1.62 (0.88, 2.97)	0.0	0.119	0	Not estimable	NA	NA
ICI resistance								
Yes	2	0.80 (0.66, 0.97)	0.0	0.026	2	0.78 (0.61, 1.00)	0.0	0.047
No	13	0.78 (0.67, 0.91)	30.8	0.001	8	0.70 (0.60, 0.82)	0.0	<0.001
ICI sequencing								
Concurrent	8	0.83 (0.73, 0.95)	49.0	0.006	6	0.73 (0.63, 0.84)	0.0	<0.001
Sequential	6	0.67 (0.49, 0.91)	0.0	0.010	5	0.67 (0.47, 0.94)	0.0	<0.001
PD-L1 level								
≥50%	2	0.74 (0.24, 2.28)	0.0	0.600				
<1%	3	0.47 (0.27, 0.81)	0.0	0.007				

Abbreviations: RT, radiotherapy; ICI, immune checkpoint inhibitor; RCT, randomized controlled trial; NRCT, Non-randomized controlled trial; PFS, Progression-free survival; OS, overall survival; mNSCLC, Metastatic non-small cell lung cancer; HR, hazard ratio; I^2^, I-squared for heterogeneity.

**TABLE 3:** Subgroup analysis of ARR and ACR of RT combined with ICIs *versus* ICIs alone in mNSCLC.

Variable	No. of trials	ARR			No. of trials	ACR		
		OR (95% CI)	I^2^ (%)	*p*-value		OR (95% CI)	I^2^ (%)	*p*-value
Type								
RCT	3	2.31 (1.19, 4.48)	11.7	0.013	3	1.85 (1.06, 3.24)	27.7	0.030
NRCT	1	1.55 (0.75, 3.23)	0.0	0.239	1	1.56 (0.47, 5.12)	0.0	0.467
RT technique								
Hypofractionated RT	3	2.92 (1.40, 6.09)	19.6	0.004				
Hyperfractionated RT	1	0.64 (0.10, 4.18)	0.0	0.640				
Traditionally fractionated RT	1	1.25 (0.24, 6.54)	0.0	0.792				
RT target								
Incranial RT	0	Not estimable	NA	NA	0	Not estimable	NA	NA
Excranial RT	3	2.31 (1.19, 4.48)	11.7	0.013	3	1.85 (1.06, 3.24)	27.7	0.030
ICI resistance								
Yes	1	0.82 (0.18, 3.71)	0.0	0.792	1	0.91 (0.33, 2.55)	0.0	0.861
No	3	2.15 (1.29, 3.60)	0.0	0.003	3	2.22 (1.24, 4.00)	0.0	0.008
PD-L1 level								
≥50%	2	1.22 (0.23, 6.38)	0.0	0.814				
1%–50%	2	6.77 (0.88, 52.27)	0.0	0.067				
<1%	2	2.80 (0.93, 8.46)	79.8	0.067				

Abbreviations: RT, radiotherapy; ICI, immune checkpoint inhibitor; RCT, randomized controlled trial; NRCT, Non-randomized controlled trial; ARR, abscopal response rate; ACR, abscopal control ratel; mNSCLC, Metastatic non-small cell lung cancer; OR, odds ratio; I2, I-squared for heterogeneity.

We classify the type of RT in which the RT dose is greater than the conventional fractionated dose of 2 Gy/fraction as hypofractionated RT. according to this criterion, When subgroup analysis based on RT dose and fraction was performed, the HRs for PFS and OS of hypofractionated RT plus ICIs were 0.95 (0.77, 1.18; *p* = 0.657) and 0.66 (0.52, 0.82, *p* < 0.001). There was no heterogeneity in the results (I^2^ was 0.0% for both). The OR of ARR was 2.92 (1.40, 6.09, *p* = 0.004). There was mild heterogeneity (I^2^ was 19.6%).

When subgroup analysis was performed according to RT target, for extracranial lesions, the HRs of PFS and OS of RT plus ICIs were 0.76 (0.66, 0.88, *p* < 0.001) and 0.72 (0.62, 0.84, *p* < 0.001), respectively. The OR value of ARR was 2.31 (1.19, 4.48, *p* = 0.013). There was mild heterogeneity in PFS and ARR (20.3%, 0.0%, and 11.7% for I^2^, respectively). For intracranial metastases, the HRs of PFS and OS for the RT plus ICIs were 0.55 (0.39, 0.79, *p* = 0.001) and 0.61 (0.38, 1.00, *p* = 0.048), respectively. There was mild heterogeneity in PFS and OS (18.3% and 10.8% for I^2^, respectively).

Subgroup analysis was conducted according to the number of RT sites. The HRs of PFS and OS of single site RT plus ICIs were 0.82 (0.72, 0.95, *p* = 0.006) and 0.74 (0.64, 0.86, *p* < 0.001), respectively. Mild heterogeneity was seen in PFS (9.2% and 0.0% for I^2^).

Subgroup analysis was done based on resistance to ICIs. In ICI-naïve patients, the HRs for PFS and OS of RT plus ICIs were 0.78 (0.67, 0.91, *p* = 0.006) and 0.70 (0.60, 0.82, *p* < 0.001). The ORs of ARR and ACR were 2.15 (1.29, 3.60, *p* = 0.003) and 2.22 (1.24, 4.00, *p* = 0.008), respectively. Mild heterogeneity was observed in PFS (I^2^ values were 30.8%, 0.0%, 0.0%, and 0.0%, respectively). For patients with immunotherapy rechallenge after ICI resistance, the HRs for PFS and OS of combination therapy were 0.80 (0.66, 0.97, *p* = 0.026) and 0.78 (0.61, 1.00, *p* = 0.047), respectively. Heterogeneity was not noted in the results (I^2^ was 0.0% for both).

Subgroup analyses were performed according to the sequencing of RT and ICIs. In the concurrent group, the HRs of PFS and OS were 0.83 (0.73, 0.95, *p* = 0.006) and 0.73 (0.63, 0.84, *p* < 0.001), respectively. There was moderate heterogeneity in PFS (49% and 0.0% I^2^, respectively). In the subgroup of ICIs after RT, the HRs of PFS and OS were 0.67 (0.49, 0.91, *p* = 0.010) and 0.67 (0.47, 0.94, *p* < 0.001), respectively. There was no heterogeneity in the results (I^2^ was 0.0% for both), regardless of whether RT was concurrent or added prior to ICIs.

Subgroup analysis was conducted according to the expression level of PD-L1. The HRs of PFS in the population with PD-L1 expression ≥50% and <1% were 0.74 (0.24, 2.28, *p* = 0.600) and 0.47 (0.27, 0.81, *p* = 0.007), respectively. There was no heterogeneity in the results (I^2^ was 0.0% for both).

### 3.3 Adverse effects of RT plus ICIs vs. ICIs alone

**FIGURE 4:**
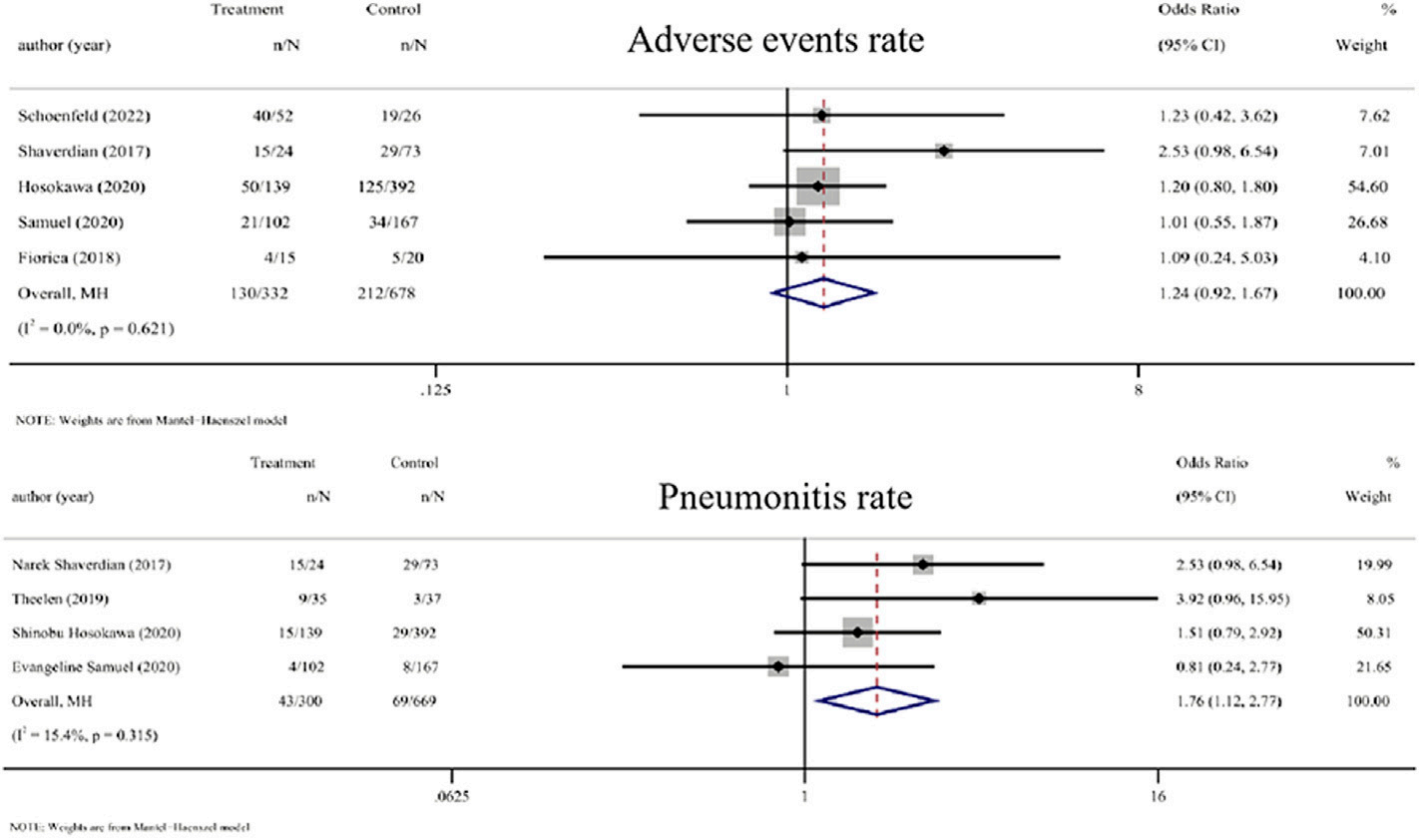
Forest plot of the adverse events rate and pneumonitis rate associated with RT combined with ICIs *versus* ICIs alone. In terms of adverse events rate and pneumonia rate, the ORs of RT combined with ICIs treatment were 1.24 (0.92, 1.67) and 1.76 (1.12, 2.77), respectively. Abbreviations: RT, radiotherapy; ICIs, immune checkpoint inhibitors; OR, odds ratio.

Five studies that compared the adverse events rate between RT plus ICIs and ICIs alone were included (Shaverdian et al., 2017; Fiorica et al., 2018; Hosokawa et al., 2021; Samuel et al., 2021; Schoenfeld et al., 2022). The OR of the adverse events rate of RT plus ICIs was 1.24 (0.92, 1.67, *p* = 0.155). Four studies that compared the pneumonia rate between RT plus ICIs and ICIs alone were included (Shaverdian et al., 2017; Theelen et al., 2019; Hosokawa et al., 2021; Samuel et al., 2021). The OR value of the pneumonia rate with RT plus ICIs was 1.76 (1.12, 2.77, *p* = 0.014). Mild heterogeneity was seen in the pneumonia rate (I^2^ = 0.0% and 15.4%, respectively) (Figure 4).

### 3.4 Sensitivity analysis and publication bias

Sensitivity analysis performed by systematically excluding each specific study from the total counts showed that the new HRs for PFS and OS and the new ORs for ARR, ACR, adverse events rate, and pneumonia rate were similar to the previously mentioned raw HRs/ORs (Supplementary Table S4). This finding signifies that any specific study could not have influenced the results of the meta-analysis.

Furthermore, statistical analysis of publication bias indicated no evidence of statistical significance (Supplementary Figures S1, S2).

## 4 Discussion

In recent years, ICIs have gained attention for the treatment of advanced solid tumors, but their efficacy is limited. Although RT can activate the tumor immune microenvironment, its immune activation and abscopal effect are obviously insufficient. However, when added to ICIs, RT can improve the long-term efficacy of the treatment and cause a considerable abscopal effect, which has been confirmed in a variety of solid tumors (Koller et al., 2017). This meta-analysis involved patients with mNSCLC, and preliminary results suggest that the addition of RT can significantly improve the long-term outcomes (PFS and OS) and abscopal local control (ARR and ACR) compared with ICIs alone. The adverse events rate was not significantly increased, but an elevation in the pneumonia rate was observed. This finding provides solid medical evidence that patients with mNSCLC can opt for RT combined with ICIs.

### 4.1 Innovations and limitations of the research

On the basis of previous studies, this meta-analysis specifically focused on mNSCLC. The effect of adding RT on long-term survival, abscopal effects, and adverse events in patients treated with ICIs was evaluated. Moreover, the effects of RT dose and fraction, RT target, the number of RT sites, previous ICI resistance, RT and ICI sequencing, and PD-L1 level on the efficacy of RT combined with ICIs were determined. Such results have rarely been reported in previous meta-analyses (Chicas-Sett et al., 2019; Fiorica et al., 2021; Geng et al., 2021).

Subgroup analysis of RT dose and fraction revealed that hypofractionated RT plus ICIs can significantly improve long-term survival as well as abscopal local control rates. Notably, the addition of hypofractionated RT did not offer a significant benefit in terms of PFS. No heterogeneity was observed in PFS only when grouped based on PD-L1 levels. Therefore, the reason for hypofractionated RT not significantly improving PFS could be that ICIs demonstrate good efficacy in mNSCLC with high PD-L1 levels. Hence, the improved efficacy of RT was overshadowed by the favorable efficacy of ICIs. The hyperfractionated RT group could not be evaluated in this study because of data limitations Hyperfractionated RT tends to alter the immune microenvironment and activate the immune cells (Barsoumian et al., 2020; Herrera et al., 2022). Extensive low-dose irradiation of metastases can activate the immune microenvironment and enhance the immune desertification environment without causing obvious adverse reactions, which has been confirmed in preclinical studies (Herrera et al., 2022). However, clinical studies are required (NCT03812549).

Subgroup analysis of the RT target showed that the addition of RT, whether extracranial or intracranial, significantly improved long-term disease control and offered survival benefits. In the case of intracranial metastases, the blood–brain barrier prevents cytotoxic T cells from entering the brain, thereby inducing tumor evasion (Quail and Joyce, 2017). The good disease control achieved in patients receiving brain RT could be attributed to the fact that RT disrupts the blood–brain barrier and results in the entry of cytotoxic T lymphocytes into the brain, thereby significantly controlling intracranial metastases (Wu et al., 2021). The results of this study indirectly support this view. In the case of extracranial metastases, different metastatic sites responded differently to ICIs, and the degree of improvement achieved with the addition of RT tended to vary. The cold immune microenvironment of liver and bone metastases limits the efficacy of ICIs (Lee et al., 2020; Zhu et al., 2022). The addition of RT can significantly improve the treatment efficacy in patients with liver metastases but not in those with bone metastases (Yu et al., 2021; Qiang et al., 2022). Owing to data limitations, only the benefit of pan-site irradiation has been discussed here. The differences in the benefits of RT for different target areas could not be examined. Among them, thoracic RT for lung metastases accounted for the majority, which could have introduced a bias.

With regard to the number of RT sites, only single-site RT combined with ICIs showed obvious benefits in terms of PFS and OS. There is insufficient data to judge whether multi-site RT is beneficial. Owing to the tumor’s specificity and site-specific immunogenicity, multi-site RT can elicit a more pronounced systemic immune response than single-site RT and is now considered to be the new paradigm (Tang et al., 2017; Brooks and Chang, 2019). Clinical studies are needed to confirm whether the efficacy of multi-site RT on ICIs is significantly higher than that of ICIs alone and whether the adverse reactions are significantly increased (Palma et al., 2019).

With regard to previous immune resistance, the addition of RT can improve PFS and OS regardless of the presence of immune resistance. However, RT cannot improve the abscopal effect of patients with immune resistance. In addition to the immune-resistant microenvironment, limited RT sites and insufficient doses of RT limit the benefits of abscopal effects (Chang et al., 2022; Ochoa-de-Olza et al., 2022).

Regarding RT and ICI sequencing, the results suggest that combined therapy can improve the efficacy of ICIs when RT is used concurrently with ICIs or when ICIs are followed by RT. RT can act both as an inducer of immune activation and a beneficiary of immune activation (Twyman-Saint Victor et al., 2015; Alsaab et al., 2017; Ochoa-de-Olza et al., 2022). Ideally, RT-induced sensitization is combined with ICI-induced immune activation. Nonetheless, the timing of RT intervention varied among the included studies; hence, the optimal timing of RT intervention could not be determined.

Subgroup analysis based on PD-L1 expression levels showed that in patients who were PD-L1-negative, the addition of RT to ICIs significantly improved the PFS compared with those who had a high PD-L1 expression. However, the survival benefit of RT based on PD-L1 expression levels could not be assessed owing to data limitations. Moreover, due to the large variety of immune checkpoint inhibitors used within each study, we were unable to obtain practically meaningful results through statistical analysis. PD-L1 is currently a biomarker that effectively predicts the efficacy of ICI. However, after RT, tumor cells release tumor-associated antigens and cytokines. Whether these can be used as predictive biomarkers to assess the degree of improvement in the efficacy of immunotherapy with the addition of RT remains to be assessed.

In terms of adverse events, previous studies have established that combined therapy does not increase the incidence of adverse events compared with ICIs alone, regardless of irradiation time and target (Sha et al., 2020; Rodríguez Plá et al., 2021). However, in terms of low-grade adverse reactions, the pneumonitis rate was significantly increased when ICIs were administered within 90 days of RT (Geng et al., 2021; Anscher et al., 2022). Our study too yielded the same result. However, data to distinguish thoracic or other metastases based on the RT target are insufficient. The occurrence of low-grade adverse events may be related to the interval between RT and ICIs; however, these events may be caused by the immune activation of RT, thereby implying a better survival prognosis (Anscher et al., 2022).

This study conducted a meta-analysis on whether the addition of RT can improve the efficacy of ICIs in the treatment of mNSCLC. The content of the discussion is rare in related studies in this field. For patients with mNSCLC who experience limited efficacy after receiving ICIs, this study has reference value regarding intervention with local RT (whether to intervene and how) as well as the screening of patients benefiting from RT. However, there are some deficiencies in this study. First, under objective conditions, the number of published RCT studies is limited. Therefore, this study may have a certain degree of bias due to the small number of RCT studies included. Controversy exists regarding radiation dose, target, number of irradiations, and intervention time. On the basis of confirming that hypofractionated RT can significantly improve the curative effect, the optimal single RT dose and the number of irradiations should be further determined. On the one hand, it is necessary to consider fully activating the immune microenvironment. On the other hand, it is imperative to avoid the occurrence of obvious adverse reactions. These reactions correlate with the pathological types of tumors, tumor size, tumor location, metastatic status, intrinsic radiosensitivity, and host characteristics. Compared with hypofractionated RT, hyperfractionation and extensive irradiation may alleviate the adverse reactions while fully activating systemic immunity. Relevant clinical studies are required. Moreover, owing to the differences in the immune microenvironment of liver, bone, and brain metastases, RT exhibits varying degrees of efficacy improvement for different metastases. Whether RT for brain metastases can obviously improve the efficacy of ICIs by damaging the blood–brain barrier needs to be supported by adequate medical evidence. For different patients, the size of the radiotherapy target volume varies with the size of the patient’s lesion. Therefore, quantitative analysis cannot be performed based on the size of the target area. The volume size of the irradiated target area also needs to be further explored to improve the efficacy of immunotherapy.There is mutual induced sensitization between RT and immunotherapy, and the period for the increase in immune cells after hypofractionated RT varies (Hettich et al., 2016). Therefore, we can use the differences in the timing of immune cell activation to intervene with ICIs. However, a shorter time interval between the two treatments resulted in an increased incidence of mild radiation pneumonitis. Hence, the optimal time for RT intervention remains to be ascertained. This study does not recommend RT for patients with high PD-L1 expression, but the number of patients included in this subgroup analysis is limited and the cutoff value of PD-L1 for the benefit of RT remains unclear. Whether predictive biomarkers other than PD-L1 and their combinations can be used for predicting the degree to which RT improves the efficacy of ICIs remains underexplored.

## 5 Conclusion

Meta-analysis of the existing data suggests that the addition of RT can significantly improve PFS and OS in patients with mNSCLC treated using ICIs. In addition, RT can significantly improve the abscopal local control of lesions outside the irradiation field *via* its immune-activating effect. Furthermore, the findings imply that the addition of RT does not increase the overall risk of adverse events, except for pneumonia.

## Data Availability

The raw data supporting the conclusion of this article will be made available by the authors, without undue reservation.

## Abbreviations

RT, radiotherapy; NSCLC, non-small cell lung cancer; SCLC, small cell lung cancer; ICIs, immune checkpoint inhibitors; RCTs, randomized controlled trials; NRCTs, non-randomized controlled trials; PFS, progression-free survival; OS, overall survival; ARR, abscopal response rate; ACR, abscopal control rate; mNSCLC, metastatic NSCLC; HR, hazard ratio; OR, odds ratio; CIs, confidence intervals.
