# Supplementary material for: Efficacy and safety of immune checkpoint inhibitors with or without radiotherapy in metastatic non-small cell lung cancer: A systematic review and meta-analysis
Source: Front Pharmacol. 2023 Jan 24;14:1064227. doi: 10.3389/fphar.2023.1064227 (PMC9902364; doi:10.3389/fphar.2023.1064227)
Supplement: Supplementary file 1 [file Table4.doc]

# Table S4. Sensitivity analyses by exclusion of each included trial.

| Exclusion of | PFS | | | Exclusion of | OS | | |
| --- | --- | --- | --- | --- | --- | --- | --- |
|  | HR (95% CI) | I^2^ (%) | P interaction |  | HR (95% CI) | I^2^ (%) | P interaction |
| Schoenfeld | 0.78 (0.69, 0.89) | 25 | ＜0.001 | Schoenfeld | 0.73 (0.64, 0.83) | 0.0 | ＜0.001 |
| Welsh | 0.80 (0.71, 0.90) | 22.3 | ＜0.001 | Welsh | 0.72 (0.63, 0.83) | 0.0 | ＜0.001 |
| Theelen | 0.79 (0.70, 0.90) | 24.7 | ＜0.001 | Theelen | 0.75 (0.64, 0.88) | 0.0 | ＜0.001 |
| Shaverdian | 0.81 (0.71, 0.91) | 15.8 | 0.001 | Shaverdian | 0.74 (0.64, 0.84) | 0.0 | ＜0.001 |
| Wang | 0.81 (0.71, 0.91) | 16.6 | ＜0.001 | Hosokawa | 0.70 (0.60, 0.81) | 0.0 | ＜0.001 |
| Hosokawa | 0.79 (0.69, 0.91) | 25.4 | 0.001 | Öjlert | 0.72 (0.63, 0.82) | 0.0 | ＜0.001 |
| Qiang | 0.77 (0.68, 0.87) | 0.0 | ＜0.001 | Samuel | 0.70 (0.60, 0.80) | 0.0 | ＜0.001 |
| Tamiya | 0.79 (0.70, 0.89) | 25.1 | ＜0.001 | Fiorica | 0.73 (0.64, 0.83) | 0.0 | ＜0.001 |
| Kataoka | 0.80 (0.71, 0.90) | 23.3 | ＜0.001 | Sheng | 0.72 (0.63, 0.82) | 0.0 | ＜0.001 |
| Öjlert | 0.79 (0.70, 0.90) | 24.6 | ＜0.001 | Guo | 0.74 (0.64, 0.84) | 0.0 | ＜0.001 |
| Samuel | 0.74 (0.65, 0.84) | 0.0 | ＜0.001 | Metro | 0.72 (0.63, 0.82) | 0.0 | ＜0.001 |
| Fiorica | 0.79 (0.70, 0.89) | 24.9 | ＜0.001 |  |  |  |  |
| Sheng | 0.79 (0.70, 0.89) | 24.2 | ＜0.001 |  |  |  |  |
| Metro | 0.79 (0.70, 0.89) | 25.4 | ＜0.001 |  |  |  |  |
| Guo | 0.81 (0.71, 0.91) | 19.4 | 0.001 |  |  |  |  |
| Exclusion of | ARR | |  | Exclusion of | ACR | |  |
|  | OR (95% CI) | I^2^ (%) | P interaction |  | OR (95% CI) | I^2^ (%) | P interaction |
| Schoenfeld | 2.15 (1.29, 3.60) | 0.0 | 0.003 | Schoenfeld | 2.22 (1.24, 4.00) | 0.0 | 0.008 |
| Welsh | 1.77 (1.03, 3.05) | 14.3 | 0.040 | Welsh | 1.65 (0.91, 3.01) | 14.6 | 0.088 |
| Theelen | 1.67 (0.95, 2.94) | 0.0 | 0.074 | Theelen | 1.49 (0.82, 2.71) | 0.0 | 0.169 |
| Wang | 2.31 (1.19, 4.48) | 11.7 | 0.013 | Wang | 1.85 (1.06, 3.24) | 19.3 | 0.026 |
| Exclusion of | Adverse events rate | | | Exclusion of | Pneumonia rate | | |
|  | OR (95% CI) | I^2^ (%) | P interaction |  | OR (95% CI) | I^2^ (%) | P interaction |
| Schoenfeld | 1.24 (0.91, 1.69) | 0.0 | 0.170 | Shaverdian | 1.57 (1.93, 2.63) | 27.6 | 0.091 |
| Shaverdian | 1.14 (0.83, 1.57) | 0.0 | 0.403 | Theelen | 1.57 (0.97, 2.55) | 4.4 | 0.069 |
| Hosokawa | 1.29 (0.83, 2.00) | 0.0 | 0.253 | Hosokawa | 2.01 (1.07, 3.78) | 37.5 | 0.031 |
| Samuel | 1.32 (0.94, 1.86) | 0.0 | 0.107 | Samuel | 2.02 (1.24, 3.30) | 0.0 | 0.005 |
| Fiorica | 1.25 (0.92, 1.69) | 0.0 | 0.153 |  |  |  |  |

# Abbreviations: PFS, Progression-free survival; OS, Overall survival; ARR, Abscopal response rate; ACR, Abscopal control rate; CI, Confidence interval; HR, Hazard ratio; OR, Odds ratio; I^2^, I-squared for heterogeneity
