# Supplementary material for: Efficacy and safety of immune checkpoint inhibitors with or without radiotherapy in metastatic non-small cell lung cancer: A systematic review and meta-analysis
Source: Front Pharmacol. 2023 Jan 24;14:1064227. doi: 10.3389/fphar.2023.1064227 (PMC9902364; doi:10.3389/fphar.2023.1064227)
Supplement: Supplementary file 2 [file Presentation2.PPT]

## Slide 1
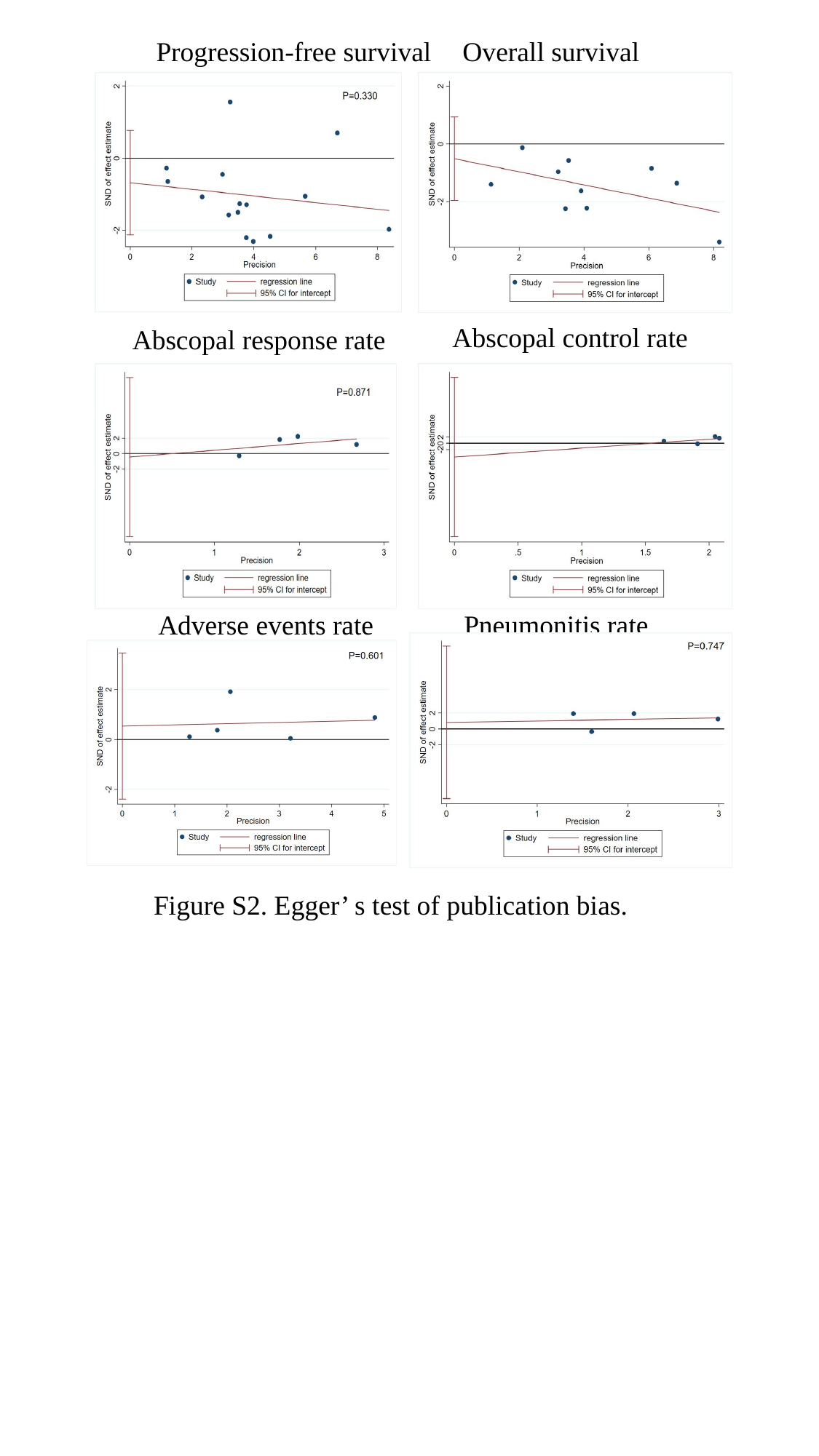

Progression-free survival
Overall survival
Abscopal control rate
Abscopal response rate
Adverse events rate
Pneumonitis rate
Figure S2. Egger’ s test of publication bias.
