# Supplementary material for: Efficacy and safety of immune checkpoint inhibitors with or without radiotherapy in metastatic non-small cell lung cancer: A systematic review and meta-analysis
Source: Front Pharmacol. 2023 Jan 24;14:1064227. doi: 10.3389/fphar.2023.1064227 (PMC9902364; doi:10.3389/fphar.2023.1064227)
Supplement: Supplementary file 4 [file Table1.DOCX]

**Supplementary literature-search methods**

Pubmed

#1 ((((((((((((((((((Radiotherapies[Title/Abstract]) OR (Radiation Therapy[Title/Abstract])) OR (Radiation Therapies[Title/Abstract])) OR (Therapies, Radiation[Title/Abstract])) OR (Therapy, Radiation[Title/Abstract])) OR (Radiation Treatment[Title/Abstract])) OR (Radiation Treatments[Title/Abstract])) OR (Treatment, Radiation[Title/Abstract])) OR (Radiotherapy, Targeted[Title/Abstract])) OR (Radiotherapies, Targeted[Title/Abstract])) OR (Targeted Radiotherapies[Title/Abstract])) OR (Targeted Radiotherapy[Title/Abstract])) OR (Targeted Radiation Therapy[Title/Abstract])) OR (Radiation Therapies, Targeted[Title/Abstract])) OR (Targeted Radiation Therapies[Title/Abstract])) OR (Therapies, Targeted Radiation[Title/Abstract])) OR (Therapy, Targeted Radiation[Title/Abstract])) OR (Radiation Therapy, Targeted[Title/Abstract])) OR ("Radiotherapy"[Mesh])

#2 ((((((((((((((((((((((((((((((((((("Immune Checkpoint Inhibitors"[Mesh]) OR (Checkpoint Inhibitors, Immune)) OR (Immune Checkpoint Inhibitor)) OR (Checkpoint Inhibitor, Immune)) OR (Immune Checkpoint Blockers)) OR (Checkpoint Blockers, Immune)) OR (Immune Checkpoint Blockade)) OR (Checkpoint Blockade, Immune)) OR (Immune Checkpoint Inhibition)) OR (Checkpoint Inhibition, Immune)) OR (PD-L1 Inhibitors)) OR (PD L1 Inhibitors)) OR (PD-L1 Inhibitor)) OR (PD L1 Inhibitor)) OR (Programmed Death-Ligand 1 Inhibitors)) OR (Programmed Death Ligand 1 Inhibitors)) OR (PD-1-PD-L1 Blockade)) OR (Blockade, PD-1-PD-L1)) OR (PD 1 PD L1 Blockade)) OR (CTLA-4 Inhibitors)) OR (CTLA 4 Inhibitors)) OR (CTLA-4 Inhibitor)) OR (CTLA 4 Inhibitor)) OR (Cytotoxic T-Lymphocyte-Associated Protein 4 Inhibitors)) OR (Cytotoxic T Lymphocyte Associated Protein 4 Inhibitors)) OR (Cytotoxic T-Lymphocyte-Associated Protein 4 Inhibitor)) OR (Cytotoxic T Lymphocyte Associated Protein 4 Inhibitor)) OR (PD-1 Inhibitors)) OR (PD 1 Inhibitors)) OR (PD-1 Inhibitor)) OR (Inhibitor, PD-1)) OR (PD 1 Inhibitor)) OR (Programmed Cell Death Protein 1 Inhibitor)) OR (Programmed Cell Death Protein 1 Inhibitors)) OR ((((((((("Immune Checkpoint Inhibitors" [Pharmacological Action]) OR (Abatacept (MeSH Term))) OR (atezolizumab (Supplementary Concept))) OR (Ipilimumab (MeSH Term))) OR (Nivolumab (MeSH Term))) OR (pembrolizumab (Supplementary Concept))) OR (relatlimab (Supplementary Concept))) OR (sotorasib (Supplementary Concept))) OR (spartalizumab (Supplementary Concept))))

#3 (((((((((((Carcinoma, Non Small Cell Lung[Title/Abstract]) OR (Carcinomas, Non-Small-Cell Lung[Title/Abstract])) OR (Lung Carcinoma, Non-Small-Cell[Title/Abstract])) OR (Lung Carcinomas, Non-Small-Cell[Title/Abstract])) OR (Non-Small-Cell Lung Carcinomas[Title/Abstract])) OR (Non-Small-Cell Lung Carcinoma[Title/Abstract])) OR (Non Small Cell Lung Carcinoma[Title/Abstract])) OR (Carcinoma, Non-Small Cell Lung[Title/Abstract])) OR (Non-Small Cell Lung Carcinoma[Title/Abstract])) OR (Non-Small Cell Lung Cancer[Title/Abstract])) OR (Nonsmall Cell Lung Cancer[Title/Abstract])) OR ("Carcinoma, Non-Small-Cell Lung"[Mesh])

#4 #1 AND #2 AND #3

Embase

#1 'immune checkpoint inhibitor'/exp

#2 'checkpoint inhibitors, immune':ab,ti

#3 'immune checkpoint inhibitor':ab,ti

#4 'checkpoint inhibitor, immune':ab,ti

#5 'immune checkpoint blockers':ab,ti

#6 'checkpoint blockers, immune':ab,ti

#7 'immune checkpoint blockade':ab,ti

#8 'checkpoint blockade, immune':ab,ti

#9 'immune checkpoint inhibition':ab,ti

#10 'checkpoint inhibition, immune':ab,ti

#11 'pd-l1 inhibitors':ab,ti

#12 'pd l1 inhibitors':ab,ti

#13 'pd-l1 inhibitor':ab,ti

#14 'pd l1 inhibitor':ab,ti

#15 'programmed death-ligand 1 inhibitors':ab,ti

#16 'programmed death ligand 1 inhibitors':ab,ti

#17 'pd-1-pd-l1 blockade':ab,ti

#18 'blockade, pd-1-pd-l1':ab,ti

#19 'pd 1 pd l1 blockade':ab,ti

#20 'ctla-4 inhibitors':ab,ti

#21 'ctla 4 inhibitors':ab,ti

#22 'ctla-4 inhibitor':ab,ti

#23 'ctla 4 inhibitor':ab,ti

#24 'cytotoxic t-lymphocyte-associated protein 4 inhibitors':ab,ti

#25 'cytotoxic t lymphocyte associated protein 4 inhibitors':ab,ti

#26 'cytotoxic t-lymphocyte-associated protein 4 inhibitor':ab,ti

#27 'cytotoxic t lymphocyte associated protein 4 inhibitor':ab,ti

#28 'pd-1 inhibitors':ab,ti

#29 'pd 1 inhibitors':ab,ti

#30 'pd-1 inhibitor':ab,ti

#31 'inhibitor, pd-1':ab,ti

#32 'pd 1 inhibitor':ab,ti

#33 'programmed cell death protein 1 inhibitors':ab,ti

#34 #1 OR #2 OR #3 OR #4 OR #5 OR #6 OR #7 OR #8 OR #9 OR #10 OR #11 OR #12 OR #13 OR #14 OR #15 OR #16 OR #17 OR #18 OR #19 OR #20 OR #21 OR #22 OR #23 OR #24 OR #25 OR #26 OR #27 OR #28 OR #29 OR #30 OR #31 OR #32 OR #33

#35 'radiotherapy'/exp

#36 'radiotherapies':ab,ti

#37 'radiation therapies':ab,ti

#38 'radiation therapy':ab,ti

#39 'therapies, radiation':ab,ti

#40 'therapy, radiation':ab,ti

#41 'radiation treatment':ab,ti

#42 'radiation treatments':ab,ti

#43 'treatment, radiation':ab,ti

#44 'radiotherapy, targeted':ab,ti

#45 'radiotherapies, targeted':ab,ti

#46 'targeted radiotherapies':ab,ti

#47 'targeted radiotherapy':ab,ti

#48 'targeted radiation therapy':ab,ti

#49 'radiation therapies, targeted':ab,ti

#50 'targeted radiation therapies':ab,ti

#51 'therapies, targeted radiation':ab,ti

#52 'therapy, targeted radiation':ab,ti

#53 'radiation therapy, targeted':ab,ti

#54 #35 OR #36 OR #37 OR #38 OR #39 OR #40 OR #41 OR #42 OR #43 OR #44 OR #45 OR #46 OR #47 OR #48 OR #49 OR #50 OR #51 OR #52 OR #53

#55 'non small cell lung cancer'/exp

#56 'carcinoma, non small cell lung':ab,ti

#57 'carcinomas, non-small-cell lung':ab,ti

#58 'lung carcinoma, non-small-cell':ab,ti

#59 'lung carcinomas, non-small-cell':ab,ti

#60 'non-small-cell lung carcinomas':ab,ti

#61 'non-small-cell lung carcinoma':ab,ti

#62 'non small cell lung carcinoma':ab,ti

#63 'carcinoma, non-small cell lung':ab,ti

#64 'non-small cell lung carcinoma':ab,ti

#65 'non-small cell lung cancer':ab,ti

#66 'nonsmall cell lung cancer':ab,ti

#67 #59 OR #60 OR #61 OR #62 OR #63 OR #64 OR #65 OR #66 OR #67 OR #68 OR #69 OR #70

#68 #34 AND #54 AND #67

Cochrane library

#1 MeSH descriptor: [Carcinoma, Non-Small-Cell Lung] explode all trees

#2 (Non-Small-Cell Lung Carcinoms):ti,ab,kw OR (Lung Carcinoma, Non-Small-Cell):ti,ab,kw OR (Carcinoma, Non Small Cell Lung):ti,ab,kw OR (Carcinoma, Non-Small Cell Lung):ti,ab,kw OR (Lung Carcinomas, Non-Small-Cell):ti,ab,kw (Word variations have been searched)

#3 (Non-Small Cell Lung Carcinoma):ti,ab,kw OR (Carcinomas, Non-Small-Cell Lung):ti,ab,kw OR (Nonsmall Cell Lung Cancer):ti,ab,kw OR (Non Small Cell Lung Carcinoma):ti,ab,kw OR (Non-Small-Cell Lung Carcinoma):ti,ab,kw (Word variations have been searched)

#4 (Non-Small Cell Lung Cancer):ti,ab,kw (Word variations have been searched)

#5 #1 OR #2 OR #3 OR #4

#6 MeSH descriptor: [Immune Checkpoint Inhibitors] explode all trees

#7 (PD L1 Inhibitors):ti,ab,kw OR (PD L1 Inhibitor):ti,ab,kw OR (PD-L1 Inhibitors):ti,ab,kw OR (Programmed Death-Ligand 1 Inhibitors):ti,ab,kw OR (PD-L1 Inhibitor):ti,ab,kw (Word variations have been searched)

#8 (Programmed Death Ligand 1 Inhibitors):ti,ab,kw OR (Checkpoint Blockers, Immune):ti,ab,kw OR (PD 1 PD L1 Blockade):ti,ab,kw (Word variations have been searched)

#9 (Checkpoint Inhibitor, Immune):ti,ab,kw OR (Checkpoint Inhibitors, Immune):ti,ab,kw OR (Immune Checkpoint Inhibitor):ti,ab,kw OR (Immune Checkpoint Blockers):ti,ab,kw OR (CTLA-4 Inhibitor):ti,ab,kw (Word variations have been searched)

#10 (Cytotoxic T-Lymphocyte-Associated Protein 4 Inhibitor):ti,ab,kw OR (Cytotoxic T-Lymphocyte-Associated Protein 4 Inhibitors):ti,ab,kw OR (CTLA 4 Inhibitor):ti,ab,kw OR (CTLA-4 Inhibitors):ti,ab,kw OR (CTLA 4 Inhibitors):ti,ab,kw (Word variations have been searched)

#11 (Cytotoxic T Lymphocyte Associated Protein 4 Inhibitor):ti,ab,kw OR (Cytotoxic T Lymphocyte Associated Protein 4 Inhibitors):ti,ab,kw OR (PD-1 Inhibitors):ti,ab,kw OR (Inhibitor, PD-1):ti,ab,kw OR (Programmed Cell Death Protein 1 Inhibitor):ti,ab,kw (Word variations have been searched)

#12 (PD 1 Inhibitor):ti,ab,kw OR (Programmed Cell Death Protein 1 Inhibitors):ti,ab,kw OR (PD 1 Inhibitors):ti,ab,kw OR (PD-1 Inhibitor):ti,ab,kw OR (Immune Checkpoint Inhibition):ti,ab,kw (Word variations have been searched)

#13 (Checkpoint Inhibition, Immune):ti,ab,kw OR (Immune Checkpoint Blockade):ti,ab,kw OR (Checkpoint Blockade, Immune):ti,ab,kw (Word variations have been searched)

#14 #6 OR #7 OR #8 OR #9 OR #10 OR #11 OR #12 OR #13

#15 MeSH descriptor: [Radiotherapy] explode all trees

#16 (Targeted Radiotherapy):ti,ab,kw OR (Targeted Radiotherapies):ti,ab,kw OR (Targeted Radiation Therapies):ti,ab,kw OR (Therapies, Targeted Radiation):ti,ab,kw OR (Therapy, Targeted Radiation):ti,ab,kw (Word variations have been searched)

#17 (Radiotherapy, Targeted):ti,ab,kw OR (Radiotherapies, Targeted):ti,ab,kw OR (Radiation Therapy, Targeted):ti,ab,kw OR (Targeted Radiation Therapy):ti,ab,kw OR (Radiation Therapies, Targeted):ti,ab,kw (Word variations have been searched)

#18 (Radiation Treatment):ti,ab,kw OR (Radiation Therapies):ti,ab,kw OR (Therapies, Radiation):ti,ab,kw OR (Treatment, Radiation):ti,ab,kw OR (Radiation Therapy):ti,ab,kw (Word variations have been searched)

#19 (Radiotherapies):ti,ab,kw OR (Radiation Treatments):ti,ab,kw OR (Therapy, Radiation):ti,ab,kw (Word variations have been searched)

#20 #15 OR #16 OR #17 OR #18 OR #19

#21 #5 AND #14 AND #20
