# Supplementary material for: Efficacy and safety of immune checkpoint inhibitors with or without radiotherapy in metastatic non-small cell lung cancer: A systematic review and meta-analysis
Source: Front Pharmacol. 2023 Jan 24;14:1064227. doi: 10.3389/fphar.2023.1064227 (PMC9902364; doi:10.3389/fphar.2023.1064227)
Supplement: Supplementary file 5 [file Table2.DOCX]

# Table S2. Quality assessment of the included randomized controlled trials

| Author | Year | Random sequence generation | Allocation concealment | Blinding of participants and personnel | Blinding of outcome assessment | Incomplete outcome data | Selective reporting | Other bias |
| --- | --- | --- | --- | --- | --- | --- | --- | --- |
| Theelen | 2019 | low risk | low risk | high risk | high risk | low risk | low risk | low risk |
| Welsh | 2020 | low risk | low risk | low risk | low risk | low risk | low risk | low risk |
| Schoenfeld | 2022 | low risk | low risk | low risk | low risk | low risk | low risk | low risk |
