# Supplementary material for: Efficacy and safety of immune checkpoint inhibitors with or without radiotherapy in metastatic non-small cell lung cancer: A systematic review and meta-analysis
Source: Front Pharmacol. 2023 Jan 24;14:1064227. doi: 10.3389/fphar.2023.1064227 (PMC9902364; doi:10.3389/fphar.2023.1064227)
Supplement: Supplementary file 6 [file Table3.DOCX]

# Table S3. Quality assessment of the included cohort studies

| Study | Selection | | | |  | Comparability |  | Outcome | | | Quality score |
| --- | --- | --- | --- | --- | --- | --- | --- | --- | --- | --- | --- |
|  | 1 | 2 | 3 | 4 |  | 1 |  | 1 | 2 | 3 |  |
| Shaverdian 2017 | * | * | * | * |  | ** |  | * | * | * | 9 |
| Qiang 2022 | * |  | * | * |  | ** |  | * | * | * | 8 |
| Wang 2021 | * | * | * | * |  | ** |  | * | * | * | 9 |
| Samuel 2020 | * | * | * | * |  | ** |  | * | * | * | 9 |
| Kataoka 2017 | * | * | * | * |  | ** |  | * | * | * | 9 |
| Hosokawa 2020 | * | * | * | * |  | ** |  | * |  |  | 7 |
| Fiorica 2018 | * | * | * | * |  | ** |  | * | * | * | 9 |
| Öjlert 2021 | * | * | * | * |  |  |  | * | * | * | 7 |
| Tamiya 2017 | * | * | * | * |  |  |  | * | * | * | 7 |
| Sheng 2021 | * | * | * | * |  | ** |  | * | * | * | 9 |
| Metro 2021 | * | * | * | * |  | ** |  | * | * | * | 9 |
| Guo 2022 | * | * | * | * |  | ** |  | * | * | * | 9 |

# Points were awarded for patient selection (maximum 4 points), the comparability of cohorts (maximum 2 points), and outcome assessments (maximum 3 points) and then summed for an overall quality rating, with a maximum of 9 points.
